# Effect of IL-1 receptor antagonist on the cerebrospinal fluid nitric oxide concentrations during experimental polymicrobial sepsis in rats

**DOI:** 10.1186/cc12992

**Published:** 2013-11-05

**Authors:** Fazal Wahab, Lucas F Tazinafo, Marcelo Eduardo Batalhão, Evelin Capellari Carnio, Maria Jose Alves da Rocha

**Affiliations:** 1 Department of Morphology, Physiology and Basic Pathology, FORP, University of São Paulo Campus de Ribeirão Preto, São Paulo, Brazil; 2 Department of General and Specialized Nursing, EERP, University of São Paulo Campus de Ribeirão Preto, São Paulo, Brazil

## Background

Recently, we observed that blocking the IL-1-IL-1r signaling pathway by central administration of IL-1ra (an IL-1 receptor antagonist) can result in increased AVP secretion and survival rate in the late phase of sepsis [1]. The mechanism of this effect of IL-1ra on AVP concentration and survival rate remains elusive. Many studies have implicated excessive production of nitric oxide (NO) as one of the important factors responsible for decreased AVP secretion during the late phase of sepsis [2]. Currently, the effect of IL-1ra on the central NO production and release during sepsis is not known.

## Materials and methods

In this study, we checked the effect of IL-1ra on sepsis-induced increased release of NO in cerebrospinal fluid (CSF). Sepsis was induced by cecal ligation and puncture (CLP). IL-1ra (9 nmol/animal) and vehicle (PBS: 2 μl/animal) were injected intracerebroventricularly to separate groups of CLP (*n *= 8/group) and control (*n *= 8/group) animals. CSF and blood samples were collected from different groups of rats (*n *= 6 to 8/group) after 1, 2, 4, 6 and 24 hours. The NO concentration in CSF was determined by chemiluminescence assay. Specific ELISA was used for AVP analysis. All experiments were carried out according to an institutional ethic committee-approved protocol (CEUA protocol number 12.1.1205.53.0).

## Results

NO levels were significantly (*P *< 0.05 to 0.005) increased in post-CLP 6-hour and 24-hour as compared with control, post-CLP 1-hour, 2-hour, and 4-hour animals. IL-1ra administration did not significantly alter the NO concentration in CSF after 1, 2, 4 and 6 hours as compared with vehicle treatment in CLP animals as well as in control. In contrast, after 24 hours NO levels were significantly (*P *< 0.02) lowered in IL-1ra-treated animals (22.36 ± 2.07 μM) as compared with vehicle-treated animals (31.97 ± 2.88 μM). The AVP concentration in IL-1ra-treated rats was significantly higher in IL-1ra-treated animals in comparison with vehicle treatment. Moreover, the survival rate of IL-1ra-treated rats was >80% while that of vehicle-treated rats was 47%.

## Conclusions

Our results have demonstrated that blocking the IL-1-IL-1r signaling pathway by central administration of IL-1ra increases AVP secretion in the late phase of sepsis, which may be beneficial for survival. We believe that one of the mechanisms for this effect of IL-1ra is through reduction of NO concentration in CSF of the septic rats.
